# cDNA Immunization of Mice with Human Thyroglobulin Generates Both Humoral and T Cell Responses: A Novel Model of Thyroid Autoimmunity

**DOI:** 10.1371/journal.pone.0019200

**Published:** 2011-04-29

**Authors:** Eric M. Jacobson, Erlinda Concepcion, Kenneth Ho, Peter Kopp, Jussara Vono Toniolo, Yaron Tomer

**Affiliations:** 1 Division of Endocrinology, University of Cincinnati College of Medicine, Cincinnati, Ohio, United States of America; 2 Division of Endocrinology, Department of Medicine, Mount Sinai School of Medicine, New York, New York, United States of America; 3 Division of Endocrinology, Metabolism, and Molecular Medicine, Feinberg School of Medicine, Northwestern University, Chicago, Illinois, United States of America; 4 James J. Peters VA Medical Center, Bronx, New York, United States of America; Cardiff University, United Kingdom

## Abstract

Thyroglobulin (Tg) represents one of the largest known self-antigens involved in autoimmunity. Numerous studies have implicated it in triggering and perpetuating the autoimmune response in autoimmune thyroid diseases (AITD). Indeed, traditional models of autoimmune thyroid disease, experimental autoimmune thyroiditis (EAT), are generated by immunizing mice with thyroglobulin protein in conjunction with an adjuvant, or by high repeated doses of Tg alone, without adjuvant. These extant models are limited in their experimental flexibility, i.e. the ability to make modifications to the Tg used in immunizations. In this study, we have immunized mice with a plasmid cDNA encoding the full-length human Tg (hTG) protein, in order to generate a model of Hashimoto's thyroiditis which is closer to the human disease and does not require adjuvants to breakdown tolerance. Human thyroglobulin cDNA was injected and subsequently electroporated into skeletal muscle using a square wave generator. Following hTg cDNA immunizations, the mice developed both B and T cell responses to Tg, albeit with no evidence of lymphocytic infiltration of the thyroid. This novel model will afford investigators the means to test various hypotheses which were unavailable with the previous EAT models, specifically the effects of hTg sequence variations on the induction of thyroiditis.

## Introduction

Autoimmune conditions targeting the thyroid are a fairly common occurrence, with a population prevalence of 1–2% [1], [2]. Hashimoto's thyroiditis (HT), the most frequent form of autoimmune thyroid disease (AITD) is an organ-specific autoimmune disease, characterized by the presence of anti-thyroglobulin (Tg) and anti-thyroid peroxidase (TPO) autoantibodies [3], [4], accompanied by a thyroidal lymphocytic infiltrate which gradually destroys the gland and, ultimately, results in clinical hypothyroidism [5]. Tg autoantibodies are a hallmark of HT, with high titers of IgG anti-Tg autoantibodies being found in >90% of HT patients [6]. The humoral attack mounted against the thyroid in HT patients is specific and progressive, as it is characterized by the presence of B cells which show increasing degrees of somatic hypermutation that produce antibodies with increasing affinities for Tg [7], [8]. Even though ∼10% of normal, healthy individuals in the United States have antibodies to Tg [9], these naturally occurring autoantibodies differ from those seen in HT patients, as they are polyreactive [10], [11], of lower affinity [12], and are predominantly IgM in isotype [6]. Overall, the etiology of HT can be described, broadly, as the interplay between susceptibility genes and environmental and epigenetic factors [13]. To better understand the pathoetiology of HT, a number of experimental systems have been advanced. To date, the ‘gold standard’ model for Hashimoto's thyroiditis, murine experimental autoimmune thyroiditis (EAT), can be induced, in susceptible mice, by immunization with either autologous or heterologous thyroglobulin, in conjunction with complete Freund's adjuvant or with lipopolysaccharide (reviewed in [14]). EAT, like its human disease counterpart, is characterized by a cellular infiltrate of the thyroid [15], as well as high titers of anti-Tg autoantibodies [16] and *in vitro* splenocyte proliferation, in response to Tg [17]. CD4+ T cells have been shown to play a pivotal role in disease induction of EAT [18]–[23]. Genetic susceptibility to EAT has been shown to occur in specific strains of mice carrying the MHC alleles H-2s, H-2k, or H-2q [24] and lies under the control of class II products of the H-2A [25] region of the murine MHC. We have recently shown that specific pocket sequences of MHC class II alleles are associated with susceptibility to EAT [26]. Moreover, the introduction of an HLA-DRA/DRB1*0301 transgene was sufficient to render a resistant strain of mouse susceptible to thyroiditis [27].

Despite the knowledge that has been gained by studying EAT and its special place in scientific history as the first model of experimentally induced autoimmunity, the method suffers from some limitations. EAT is not a model that can discriminate between subtle differences in immune responses especially to different thyroglobulin molecules. In fact we have previously shown that specific Tg amino acid variants confer susceptibility to AITD as well as EAT [28]. Although spontaneous models of autoimmune thyroiditis exist (e.g. OS (obese strain) chickens [29], praomys [30], beagles [31], NOD mice [32], and Buffalo [33] or BB/W [34] rats) that are more pathogenetically and histologically relevant to HT than EAT, none of these models has proven a practical alternative to EAT. Therefore, there is a current need for a thyroid autoimmunity model that will be amenable to testing the contribution to disease made by potential modifiers of hTg peptide presentation, while allowing breakdown of tolerance without adjuvants. In the current manuscript we report the development of an improved model, induced by an hTg cDNA vaccination, in which ‘naked’ Tg plasmid cDNA is introduced into skeletal muscle, and is presented locally to T-cells resulting in thyroid autoimmunity. Our new model shows both B and T cell responses against the introduced hTg antigen, and allows the flexibility of testing hypotheses which hitherto could not be addressed by the extant model of HT.

## Materials and Methods

### Thyroglobulin cDNA

Several overlapping fragments of the human *TG* coding sequence were generated by RT-PCR using proof reading *Pfu* polymerase (Invitrogen, Carlsbad, USA). The fragments were joined by overlap extension PCR or using unique restriction sites in the TG coding sequence. The final construct was inserted into a pcDNA3.1 plasmid (Invitrogen, Carlsbad, USA) and submitted to direct sequence analysis. The TG coding sequence contains at least 15 polymorphisms. We chose the human TG sequence reported by Van de Graaf *et al.* as a reference sequence and corrected any deviation from this sequence using site-directed mutagenesis [35].

### Preparation of Plasmid DNA

DNA was freshly prepared using the BioRad maxiprep kit. DNA was ethanol precipitated then resuspended and adjusted to a concentration of 1.5 µg/µl.

### Thyroglobulin protein

Mouse and human thyroglobulin were a gift from the laboratory of Dr. Terry F. Davies, James J. Peters VA Medical Center.

### Mice

C3H/Hen mice were obtained from Taconic laboratories. Mice which were used were exclusively female and were 8 weeks of age. All protocols were approved by the Mount Sinai School of Medicine Institutional Animal Care and Use Committee (animal use protocol #09-00149).

### cDNA Immunization Protocol (without electroporation)

Female C3H/Hen mice were injected with bupivacaine into the right *quadriceps femoris* at 2 months of age. Three days after the induction of muscle injury, mice were immunized with hTg cDNA (75 µg in 50 µl of PBS) by injection into the same area which received the bupivacaine. Subsequently, three boosts (each administered into the right *quadriceps femoris*), spaced by approximately one-week intervals, were performed. Animals were sacrificed 3 weeks after the final boost.

### cDNA immunization with electroporation protocol

All electroporation experiments were conducted under anesthesia as follows: Mice were anesthetized with Avertin (2,2,2, tribromoethanol, Sigma-Aldrich T4, 840.2). Avertin was administered i.p. at a dosage of 0.4–0.6 mg/gram of body weight. After mice were fully anesthetized, muscle was dissected and exposed. Following dissection, mice were injected into the specified muscle group with either 50 µl of 1.5 µg/µl hTg cDNA (a total of 75 µg) or 50 µl of 1.5 µg/µl of empty vector (total of 75 µg)−pcDNA3.1+ (Invitrogen). DNA was delivered in PBS/1.67% mannitol). Immediately after injection of Tg cDNA or empty vector, a current was administered to the site of injection using a square wave generator. During the exploratory phase of this work (comparison of electroporation *vs.* no electroporation) conditions were 6 pulses total, 60 V/cm, and 50 ms pulse length, with a 200 ms respite between pulses. Later, conditions were changed to 200 V/cm field strength, eight pulses total, and 20 ms per pulse cycled at 1 Hz (120 ms respite between pulses). These latter conditions were based on the methodology of Mir *et al.* [36], who reported very efficient plasmid DNA transfer in muscle fibers by using square-wave electric pulses of low field strength (less than 300 V/cm) and of long duration (more than 1 ms). We found that 3–4 cDNA immunizations/electroporations spaced by 1 week intervals gave the best results. Subsequent boosts were given to different muscle groups in each immunization as follows: (1) left *quadriceps femoris*, (2) right *quadriceps femoris*, (3) left *triceps brachii*, and (4) *right triceps brachii*. For experiments involving antibody analyses, blood samples were taken 2–3 weeks after the final boost. However, for all splenocyte-based assays (i.e. T-cell proliferation and cytokine assays), animals were sacrificed 2–3 months after the final boost, in order to study the long-term responses of the electroporation-based cDNA strategy.

### Classical EAT protocol

C3H/HeN mice received 50 µl of human Tg in 100 µl of PBS. This Tg was administered through a tail vein. 2–3 hours after the Tg, 20 µl LPS was administered. Three immunizations, spaced by approximately two week intervals, were administered. Animals were sacrificed two weeks after the third and final round of immunization.

### Lymphocyte isolation

Spleens were collected from mice and kept on ice in Hanks Balanced Salt Solution (HBSS). To harvest lymphocytes, the spleens were placed in complete RPMI medium [RPMI 1640 medium supplemented with fetal bovine serum (FBS; 10%), L-glutamine (2 mM) and penicillin-streptomycin (100 U/ml, 100 µg/ml)] (Hyclone, Fisher Scientific), and cut in several places. These were then pressed in a circular motion with a plunger from a 6 ml syringe until only fibrous tissue remained. To further disperse clumps in the suspension, it was drawn up and expelled several times through a 6 ml syringe using a 19 gauge needle, and then the suspension was filtered twice through a nylon screen (70 µm cell strainer) into a 50 ml falcon tube. The cell suspension was then spun for 10 minutes at 200× g and the supernatant was discarded. Cells were again suspended in complete RPMI medium and centrifuged a second time, and the media were discarded. To remove remaining non-lymphocytic cells from spleen, 5 ml of ACK lysis buffer was added to the cells for 5 minutes, after which complete RPMI medium was added to fill the tube, and centrifuged for 10 minutes. The remaining cell pellet was washed and resuspended in 10 ml of complete RPMI medium for counting and plating.

### T-cell proliferation assays

Cells isolated from spleens of mice were cultured in RPMI 1640 medium supplemented with fetal bovine serum (FBS; 10%), L-glutamine (2 mM) and penicillin-streptomycin (100 U/ml, 100 µg/ml) (Hyclone, Fisher Scientific). Cells were plated 2×10^5^ cells per well in medium in a total volume of 200 µl. Cells were treated with either PBS (negative control), thyroglobulin (40 µg/ml) or concavalin A (2 µg/ml; positive control). After 48 hrs, cells were pulsed with 1 µCi/well of [^3^H]thymidine (MP biomedical, Costa Mesa, CA). Cells were harvested 18 h later, and [^3^H]thymidine incorporation was measured in a scintillation counter (TopCount·NXT™; PE life sciences, Boston, MA). All assays were performed in quadruplicates. Data are expressed as stimulation index. We calculated the stimulation index by using the following formula: Stimulation index = (CPM of the antigen treated lymphocytes−background)/(CPM of PBS treated lymphocytes−background).

### ELISA assay for the detection of thyroglobulin antibodies

10 µg/ml of mouse thyroglobulin in carbonate buffer (pH 9.6) (total volume 100 µl) was used to coat wells of an ELISA plate, and incubated overnight at 4°C. The plate was washed 4 times with PBST (PBS+0.05% tween). 200 µl of blocking buffer (PBST+2.5% bovine serum albumin) was added to wells and incubated for 1 hour at 37°C. The plate was then washed 4–6 times with PBST. Sera were diluted in 1∶100 in PBST/1%BSA and added in a total volume of 100 µl, in triplicates. Binding was allowed to proceed for 2 hours at room temperature. Unbound sera were removed by washing 4–6 times with PBST. Then 100 µl of goat anti-mouse IgG-HRP (Sigma-Aldrich), diluted 1∶500 in PBST/1% BSA was added. The binding was allowed to proceed for 30 minutes at 37°C. Unbound antibody was removed by washing with PBST 4 times. 100 µl of freshly prepared PNPP substrate was added to each well. Readings were performed by an ELISA reader at 405 nm.

### Isotype concentration quantification

In order to test for the isotype of the detected anti-Tg antibodies, a modified version of the methodology described by Chronopoulou *et al.* [37] was employed. In brief, a 10 µg/ml solution of mouse thyroglobulin in pH 9.6 carbonate buffer was used to coat ELISA plate wells as described above. The plate was washed 4 times with PBST (PBS+0.05% Tween). After the final wash, 200 µl of blocking solution (PBST+2.5% BSA) was added to each well. Blocking was performed for 1 hour at 37°C. The plate was washed 6 times with PBST. 100 µl of diluted mouse sera (1∶100 in PBST+1% BSA) was added to each well. Incubation proceeded for 1 hr at 37°C. Unbound sera were removed by washing 10 times with PBST. Next, 100 µl of specific anti-mouse IgG subclass conjugated to alkaline phosphatase (diluted 1∶5000 in PBST 1% BSA) were added and incubated at 37°C for 30 minutes. Subclass specific antibodies could detect mouse IgG_1_, IgG_2a_, and IgG_2b_ (Caltag, catalog # M32108, M32308, M32508). Excess subclass-specific antibody was removed by washing 10 times with PBST. For detection of signal, 100 µl of freshly prepared PNPP substrate (2 tablets/10 ml of 10 mM diethanolamine solution) was added. Signal was read using an ELISA plate reader at 405 nm.

In order to calculate the amount of a specific IgG subclasses in the sera, a serum with known amounts of IgG subclasses (MP Biomedicals catalog #64091) was employed as follows: on the same plate which was used to read the immunized mouse sera, a series of wells were coated with anti-mouse IgG (Sigma M6898), diluted to 50 µg/ml, in the carbonate pH 9.6 buffer and was added at 100 µl per well. Washing was performed as described above. The standard reference serum was diluted 1∶10 in PBST to establish a working solution. Next, 7 serial dilutions of reference serum solution were performed (1∶5, 1∶25, 1∶125, 1∶625, 1∶3125, 1∶15625, and 1∶78,125). 100 µl of diluted reference serum was added to the wells coated with anti-mouse IgG. These reference dilutions were performed in triplicate to construct the calibration curve. Standard curves typically gave an R^2^≥0.95.

### Cytokine ELISAs

Interferon gamma and interleukin 4 levels in the supernatant of stimulated lymphocytes were determined by a commercial ELISA kits (BD Biosciences, OptEIA™).

### Statistical Analyses

The differences between T cell proliferation indexes and antibody levels at different conditions were analyzed using Student's t-test. P-values of <0.05 were considered significant.

## Results

### Electroporation increases autoimmune responses to Tg

**Figure 1:**
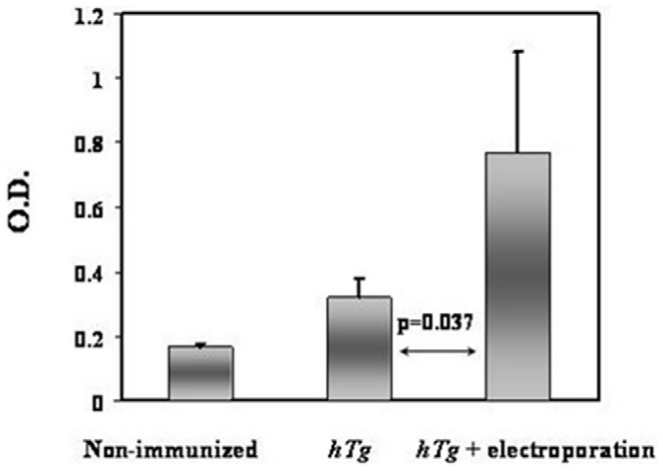
Effects of different means of cDNA delivery on anti-Tg antibody response. Shown is an ELISA measuring total IgG (all subclasses), directed against murine thyroglobulin (Tg). The results demonstrate a significant anti-Tg response in mice immunized with hTg cDNA. For this analysis, we compared 2 non-immunized mice, the 2 best responders, out of a group of 6 that were immunized with hTg without electroporation, and 6 hTg cDNA immunized/electroporated mice. Relative to directly immunized mice, the use of an electric current (6 pulses total, 60 V/cm, and 50 ms pulse length, with a 200 ms respite between pulses) caused a significant increase in the level of anti-Tg antibodies (p = 0.037). All readings were performed in triplicate. Data are shown as mean + standard error of the mean (SEM). Animals immunized with empty vector (pCDNA3.1+) exhibited no response (data not shown) to mouse Tg.

Despite the immense potential of skeletal muscle to uptake and express a multitude of plasmid cDNAs [38], the inherent inefficiency of this process results in low levels of target gene expression and immune responses [39], [40]. Hence, a number of methodologies have been advanced to increase the efficiency of cDNA uptake. Muscle regeneration, induced by treatment with cardiotoxin or bupivacaine, prior to cDNA immunizations, has been shown to increase target gene expression by up to 80-fold [41]. Similarly, electroporation of skeletal muscle immediately after the injection of cDNA has been demonstrated to result in a significant improvement in transfection efficiency [42]. In order to assess the effects of different potential modes of hTg cDNA immunization delivery, we employed two approaches, one direct, and the other facilitated by electroporation. We injected, in 3 rounds, *hTg* plasmid into the muscles of 8-week old female C3H/HeN mice that had been previously treated with bupivacaine, and compared the resultant anti-Tg antibody responses to those of mice that received hTg immunization in conjunction with electroporation. Mouse sera were analyzed for the presence of anti-murine Tg IgG. Comparison of sera from the two groups of mice showed that electroporation-facilitated cDNA delivery significantly increased the levels of Tg reactive antibodies (**Figure 1**) compared to direct delivery in regenerating muscle. In view of these initial results all subsequent experiments were performed with electroporation.

### hTg cDNA immunization and electroporation induces T-cell proliferative responses to Tg

**Figure 2:**
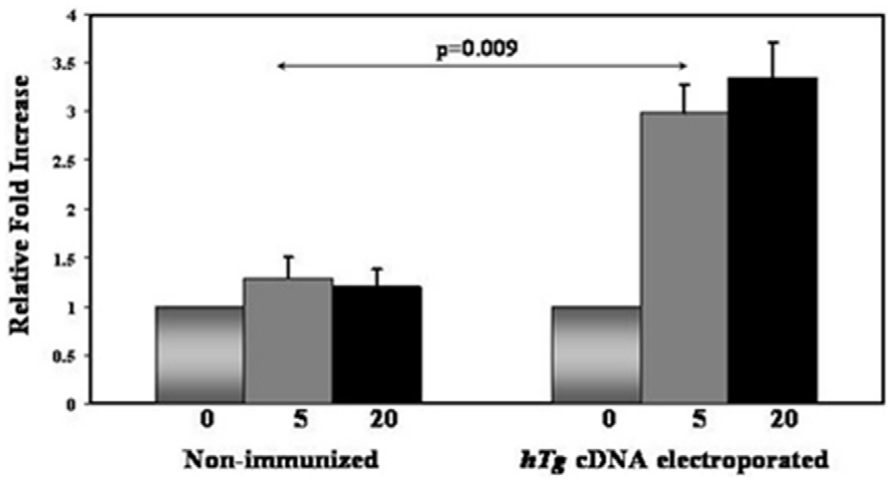
T cell stimulation. Splenocytes isolated from immunized (2–3 months after the final boost) and non-immunized C3H/HeNmice were incubated with increasing concentrations of hTg protein for 72 hrs. Splenocytes from hTg immunized/electroporated mice showed significantly increased proliferation, compared to similarly challenged splenocytes from non-immunized mice. The x axis represents the concentration (µg/ml) of hTg which was used to stimulate the splenocytes. All T-cell proliferation experiments were performed in triplicates. Data, which were initially recorded as cpm, were subsequently expressed relative to splenocytes which did not receive thyroglobulin, are shown as mean + SEM.

Splenocytes isolated from *hTg* injected and electroporated C3H/HeN female mice proliferated in response to *hTg*. Splenocytes from electroporated mice showed significantly increased proliferation, compared to similarly treated splenocytes from non-immunized mice (**Figure 2**).

### A potential Th1 bias of the T-cell response in hTg immunized/electroporated mice

**Figure 3:**
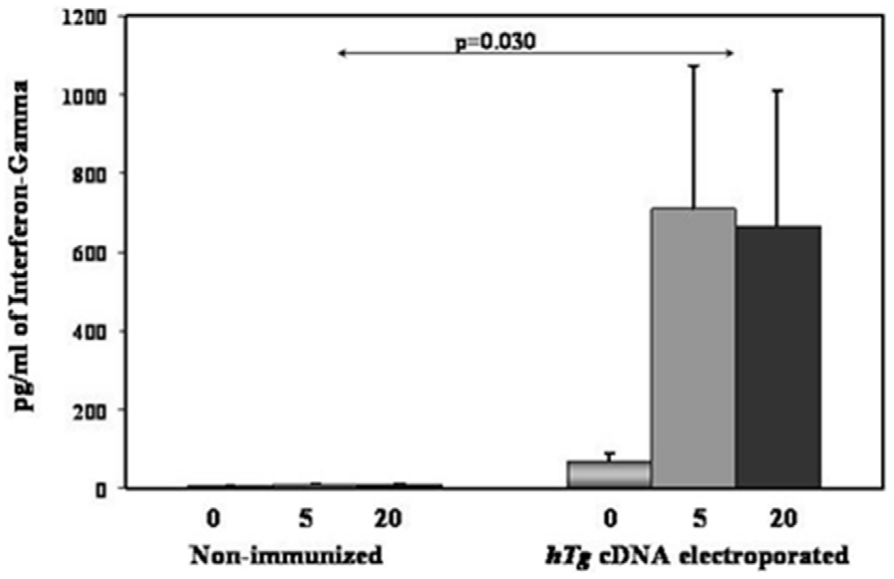
Th1 nature of the Cytokine Profile. Splenocytes were isolated from immunized/electroporated (2–3 months after the final boost) and non-immunized mice. 2×10^5^ cells were challenged with 0, 5, or 20 µg/ml of human thyroglobulin. Supernatants were collected and were analyzed for the presence of cytokines. hTg-treated splenocytes from immunized/electroporated animals secreted significantly higher levels of interferon-gamma than controls. Mice immunized with empty vector showed no differences compared to the non-immunized controls (data not shown). All readings were performed in triplicate. Data are shown as mean + SEM.

To determine if cDNA immunization with hTG resulted in a Th1 or Th2 polarization of the anti-Tg response we further characterized the T cell responses by examining cytokine production. IFN-gamma production was significantly increased in response to hTg cDNA (**Figure 3**), while IL-4 was below the limits of detection (data not shown). Hence, the anti-hTg immune response in our mice is suggestive of a Th1 bias, but does not rule out involvement of the TH17 pathway.

### Analysis of the antibody response to cDNA immunization/electroporation with hTg

**Figure 4:**
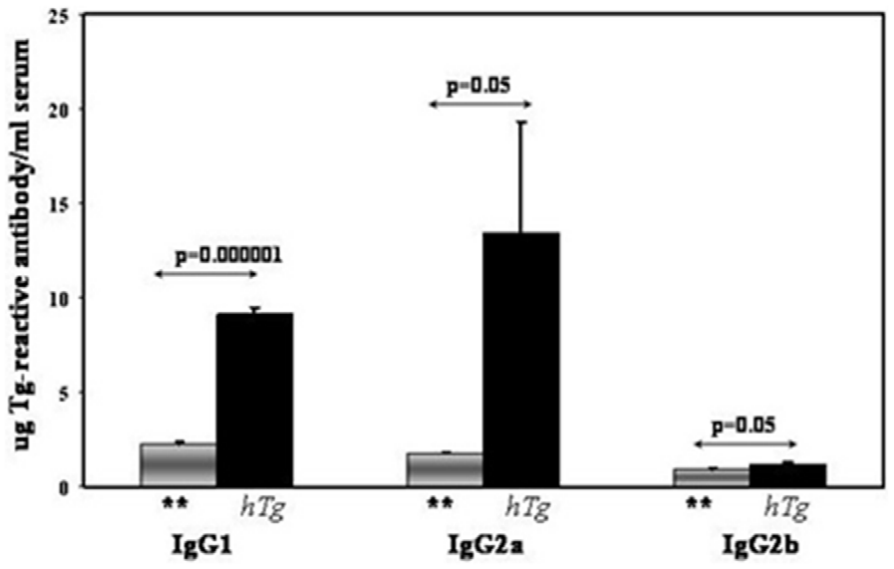
IgG subtypes or anti-mTg antibodies produced by hTg immunized/electroporated mice. Three, 8-week old C3H/HeN mice received hTg immunization/electroporation while 4 mice received an empty pcDNA plasmid electroporation (denoted by ‘**’). Anti-mouse Tg antibodies of the IgG2a subclass were the most prevalent isotype, followed by IgG1. There were very low levels of anti-mTg antibodies of the IgG2b class. All ELISA experiments were performed in triplicates. Data are presented as mean + SEM.

All the mice that were immunized with hTg cDNA and electroporated developed a robust antibody response to mouse thyroglobulin (mTg) demonstrating the development of a *de novo* thyroid autoimmunity. Analysis of the IgG subtype elicited by cDNA immunization/electroporation with Tg is further suggestive of the Th1 predominance of the immune response to Tg observed by the cytokine profile. IgG2a showed the highest levels among the anti-hTg IgG subtypes, suggesting a Th1 bias of the autoimmune response (**Figure 4**).

### Induction of the classical EAT model

**Figure 5:**
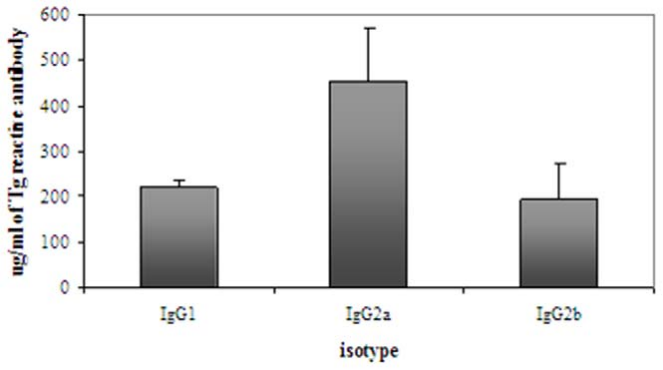
IgG subtypes or anti-mTg antibodies produced by C3H/HeN mice induced with classical EAT by immunizing with hTg protein and adjuvant. Three, 8-week old C3H/HeN mice were intravenously immunized with 50 µg of hTg in 100 µl of PBS into the tail vein. 2–3 hours later, 20 µl of LPS was injected into the tail vein. Three immunizations, spaced two weeks apart, were administered. Animals were sacrificed two weeks after the third and final round of immunization. At sacrifice, sera were collected and were analyzed for the presence of anti-mTg and the specific isotype of the antibodies. All ELISA experiments were performed in triplicates. Data are shown as mean + SEM.

In order to compare our new model to the classical EAT model, we also induced classical EAT in the mice by immunizing them i.v. with hTg protein followed by i.v. LPS 2–3 hrs later. As expected, we saw a robust anti-Tg antibody response. Intriguingly, the classical EAT model showed a different IgG subtype response than the cDNA immunized/electroporated mice. While IgG2a showed high levels in the classical EAT similar to the cDNA immunization model, IgG2b showed very high levels in the classical EAT, while its levels remained very low in the cDNA immunized mice (**Figure 5**).

### Thyroid Histology

Hematoxylin-eosin staining of thin sections from thyroid glands of mice from the different experiments did not show any evidence of lymphocytic infiltration into the thyroid gland (data not shown).

## Discussion

Several cDNA vaccine-based animal models have been recently developed, the most notable of which is experimental autoimmune Graves' disease (EAGD), induced by immunization of BALB/c mice with an Adenovirus vector carrying the TSH receptor [43]. The main advantage of cDNA immunization based mouse models is that they are easily amenable to genetic manipulations of the cDNA immunogen. For example, in the case of EAGD, it was found that immunization with the alpha subunit of the TSHR alone gave a stronger immune response and more severe disease phenotype than when using the full-length TSHR [44], [45]. Moreover, since the auto-antigen is presented in the correct conformation during cDNA immunization (as it is produced and expressed on muscle cells) the immune response generated by cDNA immunization is more relevant to the human version of disease.

Up until 1980, it was believed that Tg reactive T-cells were completely deleted by central tolerance mechanisms. Therefore, the dogma was that EAT can only be induced with adjuvant which stimulated non-Tg reactive T-cells to bypass the tolerance to Tg and provide help for Tg-reactive B-cells. However, in 1980–1981, Rose and colleagues published seminal studies clearly demonstrating that repeated immunizations with high doses of Tg without adjuvant could induce a strong anti-Tg antibody response and lymphocytic infiltration of the thyroid [46], [47]. These studies refuted the dogma and showed clearly that Tg autoreactive T-cells do escape central tolerance. Moreover, the authors correctly predicted the existence of peripheral regulatory mechanisms for these auto-reactive T-cells that escaped tolerance. Indeed, McLachlan *et al.* have recently shown that depletion of Tregs by itself is enough to cause anti-Tg and anti-TPO antibody response and massive lymphocytic infiltration of the thyroid when mice were immunized with TSHR cDNA with no adjuvant [48], [49]. Along a similar vein of thought, our manuscript also supports the hypothesis that anti-Tg reactive T-cells escape central tolerance, since our model shows the induction of an immune response without the addition of adjuvant.

Traditionally, cDNA vaccines are best known as agents that can generate protective immunity against infectious disease and experimental cancer, or can supply a therapeutic dose of a gene product (reviewed in [39]). However, the same principles which allow DNA vaccination to produce a prolonged, endogenous expression of antigen [50], along with the generation of long-term humoral and cellular immune responses [51]–[53], could be utilized to promote and sustain an immune response against a self-antigen, that is to create a specific autoimmune condition. Mechanistically, the immunostimulatory nature of the plasmid DNA itself, due to CpG motifs, directs the de novo synthesis of MHC II molecules and chemokines (e.g. monocyte chemotactic protein-1), allowing the myocyte to become an active participant in the development of an immune response by presenting the autoantigen. Indeed, in terms of breaking peripheral tolerance to the thyroid, cDNA-based vaccines have actually proven to be more accurate disease models than traditional protein/adjuvant-based autoimmune models. For example, the administration of TPO in a plasmid format, resulted in high affinity antibodies with limited epitopic recognition, similar to the TPO antibodies produced in HT patients [54]. Subsequent manipulation of this protocol produced lymphocytic infiltration in DR3+ MHCII-null/NOD mice, creating an even more accurate model of disease [55]. Finally, the EAGD model, in which *TSHR* cDNA is administered in an adenoviral vector, produces TSHR stimulating antibodies and hyperthyroidism [43]. Hence, we anticipated that an optimized HT cDNA model, using thyroglobulin cDNA expressed in the microenvironment of the mouse myocyte, will be sufficient to initiate an immune response to Tg.

Thyroglobulin is a 660 kDA homodimeric, iodinated glycoprotein that serves as a precursor for thyroid hormones [15], and represents a remarkable 75%–80% of total thyroidal protein [56]. Two whole genome linkage screens have shown strong evidence of linkage of HT with the Tg locus on 8q24 [57], [58]. Furthermore, case-control and family-based association studies, using microsatellites in introns 10 and 27 of the Tg gene demonstrated association of the *Tg* gene with HT [59]. Hence, it is likely that Tg is the primary antigen in HT precipitating the disease, even though anti-TPO antibodies are the most specific antibodies for clinical diagnosis. Indeed, recent data support the notion that Tg is the primary antigen precipitating AITD and breaking tolerance [60], [61]. Therefore, selecting Tg for cDNA immunization was a logical choice, in order to precipitate a break in tolerance to the thyroid. Our study demonstrated that electroporation in addition to hTg cDNA immunization significantly increased the immune response elicited by Tg. Electroporation consists of applying an electric potential, usually in the form of a square wave [62], to an area of muscle that has been vaccinated with cDNA, and has been advanced as a means to ensure that foreign DNA is taken up more efficiently and expressed for longer durations [63]–[65].

The current new model we have developed demonstrates that it is possible to breakdown tolerance to Tg, using cDNA immunization coupled with electroporation. However, despite the breakdown in tolerance, the mice did not develop lymphocytic infiltration of the thyroid and hypothyroidism. One potential explanation for the lack of lymphocytic infiltration of the thyroid is that we have immunized with human Tg. Even though there is 70% amino acid sequence identity between human and mouse Tg, enough differences may exist that it may not be sufficient, using *hTg* cDNA in mice, to induce a response to mouse Tg coupled with infiltration of the thyroid. A similar situation is seen in the cDNA immunization-based model of Graves disease (EAGD). In the EAGD model, which uses cDNA immunization with human TSHR to induce Graves' disease, a significant T-cell and antibody response is observed to human TSHR, and the antibodies, through cross-reaction with the mouse TSHR, stimulate the mouse TSHR causing thyrotoxicosis. Nevertheless, there is no T-cell infiltration of the thyroid [43]. Attempts to induce EAGD with mouse TSHR resulted in no autoimmune response at all. The suggested reason for the inability to breakdown tolerance when immunizing with mouse TSHR was that these mice have inborn central tolerance to mouse self-antigens including mouse TSHR [66]. It is possible that a similar phenomenon would be observed in our model, if one tried to induce EAT by immunization with mouse Tg cDNA.

Despite the lack of lymphocytic infiltration of the thyroid, our findings furnish significant implications and afford new options to future studies focusing on the etiology of AITD. The main advantage of our model is that it enables immunization with human Tg and it facilitates manipulating the sequence of the hTg cDNA (through site directed mutagenesis), in order to investigate the importance of different parts of the molecule in inducing autoimmunity. Indeed, our genetic studies have demonstrated a strong association between amino acid variants in the thyroglobulin gene and AITD, suggesting that subtle sequence changes in hTg may have a significant effect on disease susceptibility [28]. These findings were in agreement with older studies by Rose and colleagues that have demonstrated that the source of Tg used to induce EAT determined the severity of disease [67]. Our novel mouse model offers the first opportunity to test the effects of the human Tg sequence on the induction of thyroid autoimmunity in mice in vivo. The thyroglobulin used in our cDNA immunizations was able to breakdown tolerance even though it was not iodinated, since it was produced in muscle cells that lack the different enzymes and transporters required for iodination of Tg. These data support the notion that iodination of Tg may not be essential for its immunoreactivity. However, it is possible that the lack of iodination was the cause of lack of lymphocytic infiltration of the thyroid. If this is the case, it would imply that iodination is not required for Tg peptide presentation and stimulation of a T-cell and B-cell response to Tg. However, iodination of Tg may be required for stimulation of cytotoxic T-cell stimulation (through MHC class I) leading to infiltration of the thyroid gland.

In summary, for the first time we have produced a mouse model of thyroid autoimmunity using cDNA immunization, coupled with electroporation, without the need for a strong adjuvant such as CFA or LPS. Our novel mouse model is unique because it involves immunization by intramuscular injection of Tg cDNA resulting in production of non-iodinated Tg in muscle cells. Because we utilized cDNA immunization our model is amenable to genetic modification of Tg used in the immunization. This model should prove useful for future studies on the role of different Tg sequence variants or Tg domains in the induction of thyroid autoimmunity. Furthermore, the milieu afforded by the mouse myocyte will allow testing, through the co-immunization and electroporation of their respective cDNA's, of the role of pro-inflammatory and pro-apoptotic factors in the pathoetiology of Hashimoto thyroiditis. Overall, this new model of thyroid autoimmunity is a model with much potential and flexibility.
